# Measles virus: Continued outbreaks while striving for eradication

**DOI:** 10.1080/21505594.2024.2386022

**Published:** 2024-08-02

**Authors:** David Jesse Sanchez

**Affiliations:** Pharmaceutical Sciences Department, Western University of Health Sciences, Pomona, California, USA

As 2024 progresses, there has been signs of increased numbers of outbreaks of measles virus infections throughout the world. From 5 April 2024 to 25 July 2024, the U.S. Centers for Disease control reported that the country moved from 113 measles cases to 188 measles cases being reported throughout the country in a diverse collection of states with most of these cases starting from three different outbreaks [1]. In addition, South Sudan reported that they are experiencing a tremendous burden of measles infection with more than 12,000 cases in the first part of 2024 [2]. This is compounded with the 2019 notice that several countries in Europe including the UK have lost their status of having eradicated measles virus and recent reports of outbreaks have been increasing [3]. These reports are just pieces of the larger picture of measles virus infection throughout the globe which the World Health Organization estimates to have been reflected in about 9 million cases of measles virus infection with 136,000 deaths in 2022. For a current perspective, Figure 1A shows a map of the reported measles cases throughout the globe so far in 2024.

**Figure 1.**
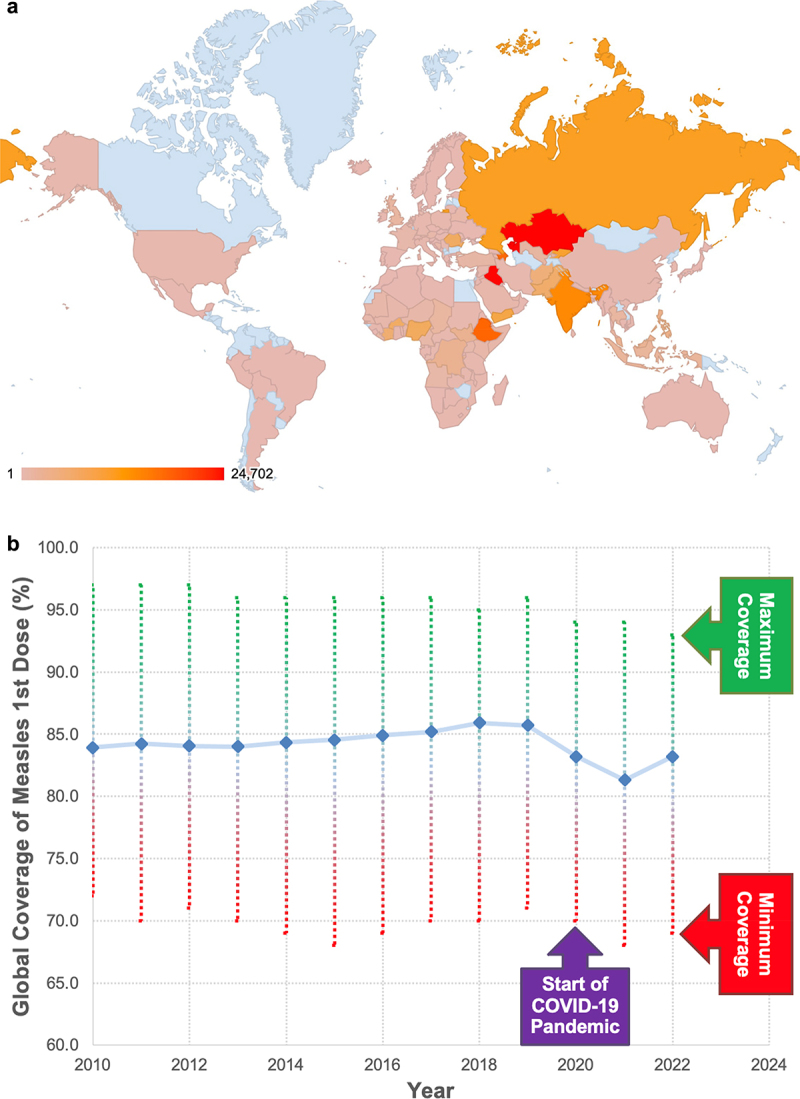
Global measles virus infection versus vaccine coverage.

Data from the World Health Organization show the level of measles virus infection versus global vaccine coverage against measles virus infection which has stayed at about 85% until the COVID-19 pandemic.

(A) A geographic heat map was generated based on data from the WHO of the total number of reported measles virus infection for January through May of 2024. Blue countries had no reported cases of measles virus. The heat map legend identifies countries with at least 1 measles virus infection so far in 2024. Of note is the global prevalence of measles virus infections.

(B) Data plotted is the percent of total number of first dose of measles virus divided by the estimated number of individuals that could have gotten their first measles virus vaccination dose in that year. The COVID-19 pandemic induced a decrease in global uptake of individuals first measles virus vaccination by almost 5% over 2 years. When taking into account that the herd immunity threshold for measles virus infection is over 90%, this shows that most of the globe has regions that are prone to outbreaks and need significant support to increase measles vaccine coverage.

## Diminishing vaccine coverage impacts herd immunity potentials

There are multiple factors that could be inducing this current surge in measles cases and outbreaks. A major challenge that has become a global concern is the lessening coverage of our population by the measles virus vaccine. The impact of the MMR (measles-mumps-rubella) vaccine on global healthcare cannot be overstated. Prior to the development of effective vaccinations to prevent measles virus infection, measles was common throughout the globe [4]. Development of an effective vaccination against measles virus infection was important work of the 1960s and vaccination against measles virus has become a common part of childhood vaccination schedules throughout the globe. The introduction of the measles virus vaccination drastically lowered the global burden of measles virus infection from more than 135 million annual cases down to the reported 9 million [4].

However, measles virus is a standout virus from the public health perspective for one important reason when discussing vaccine coverage: the R_0_. R_0_ or the reproduction number for measles virus infection is the number of contacts that are predicted to acquire an infection from one person with the viral infection. For measles virus, that number has been consistently estimated to be between 12 and 18 [5]. This is in sharp contrast to the R_0_ for different influenza virus strains or variants of SARS-CoV-2 which all range between 1 and 2 [6,7]. Even if smallpox virus is reintroduced into our society, it is currently modelled to spread with an R_0_ at about 5, still only a fraction of the R_0_ for measles virus infection [8]. What does that mean for vaccination coverage? The higher the R_0_, the higher the vaccine coverage has to be to protect a community against outbreaks or spread from imported cases of the virus. The herd immunity threshold is calculated with (*R*_0_ −1)/*R*_0_ showing that for a R_0_ of 2, 50% vaccine coverage of the population is sufficient to provide some herd immunity benefit. However, for a virus like measles with an R_0_ of about 15, the required coverage jumps to more than 90% [9]. The US CDC estimates global coverage for the measles vaccine to be at 74%.

In 2000, the US declared measles eliminated from their country due to consistently low number of cases. However, vaccine hesitancy and the lack of immediate threat of this viral infection has impacted overall measles vaccine coverage in the US. Some have gone so far as to dismiss the health benefit of measles virus vaccination by claiming natural infection is superior to vaccinations. However, natural infection by measles virus has been found to dramatically reduce the antibody repertoire in human with reduction as high as 73% of antibody recognition potential [10]. In contrast, that group showed that there was no similar loss to antibody recognition potential of a cohort following MMR vaccination highlighting the different aspects of vaccination against measles virus infection.

This diverse landscape of vaccine coverage has made movement towards eradication hard to turn to reality. Models have been developed in an effort to predict how to get to eradication of measles virus and few offer a path that does not involved significant investments in resources [11]. The eradication of a viral pathogen is classically dependent on two major factors: is the virus restricted to human tropism with no reservoirs and does the virus have a visible, acute infection with notable signs and symptoms. Smallpox virus infection had those two factors and a global plan to eradicate the virus from natural infections can be seen as a major triumph of public health and medicine of the 1900s [12]. While viruses like SARS-CoV-2 and Influenza A Virus do not check off the two key factors for eradication, measles virus is ideally setup as a virus that can be eradicated. However, the trend seems to be countries losing measles eradication status, not gaining it.

## Is eradication of measles attainable?

So why do measles virus infections continue to evade plans for eradication? Without a more focused global response to support the medical systems in regions where measles virus infection is endemic, there exists an unbalanced burden of global measles virus infection [13]. The balance between countries maintaining the status of having eliminated measles virus versus the movement of some countries from a declaration of eliminated measles virus back to endemic spread of measles virus continues to push back against measles virus eradication. Of critical importance was the finding that in 2021, 81% of children received their first dose of the measles vaccine which was the lowest coverage of this population since 2008 [13].

Countries like the US that have long since had low rates of measles virus infection now have signs of increasing infection in unvaccinated populations. In the cases reported to the US CDC, it is estimated that 81% of the cases are in individuals that are unvaccinated or with unknown vaccination status, and the majority of these cases are in children. The model for these outbreaks in regions has becomes that travel between endemic regions and areas with little to no infection can bring infection that leads to outbreaks if immunization rates in the nonendemic regions drop below the herd immunity thresholds. These lower rates can be in small, local communities where infections can rapidly spread amongst close contacts as was seen in a local outbreak in 2016 in the US, however, rapid public health responses quickly stopped further spread of the virus [14]. The need and effectiveness for surveillance and rapid respond to control the spread of measles virus infection brought into countries that have declared measles virus eradicated was highlighted during the resettlement of Afghan evacuees in the US in 2021 [15].

## COVID-19 pandemic directly stopped vaccine distribution

While lingering vaccine hesitancy and lack of access to vaccination continue to block eradication, we also exist where the impact of the COVID-19 pandemic continues to impact public health. During the height of the pandemic in 2020, more children missed their first dose of the measles vaccine than in two decades before [16]. The ramification of this decrease is expected to spread as lower vaccine coverage invites outbreaks throughout global locations. As shown in Figure 1B, the vaccine uptake during the past years, while consistent is still below measles virus the herd immunity threshold. Importantly, the impact of COVID-19 is highlighted by the lower vaccine uptake during the height of the pandemic.

## Where do we go now?

Measles eradication is still attainable and is not an impossible task. The best tool in our approach is the measles virus vaccine. Increasing vaccine coverage and responding to outbreaks with increased urgency towards the goal of eradication is required. As much as many places have pushed measles virus into a lower tier of health priorities, it is one that we as a global community can still directly work on towards an attainable goal.

## Data Availability

Data for Figure 1 were obtained from the World Health Organization and processed in Microsoft Excel. All data and excel files used for this figure are freely available from WHO at https://www.who.int/data/gho/data/indicators/indicator-details/GHO/measles—number-of-reported-cases for Figure 1A and https://data.who.int/indicators/i/BB4567B for Figure 1B and also from the author’s Open Science Framework site (https://osf.io/wyfmk/?view_only=20650d39fe0b4178bdb00ec3792f39dd) where the downloaded and processed data used to produce Figure 1 is available. Variant Figures are also included for further visualization.
